# Population genetic correlates of declining transmission in a human pathogen

**DOI:** 10.1111/mec.12099

**Published:** 2012-11-02

**Authors:** Standwell C Nkhoma, Shalini Nair, Salma Al-Saai, Elizabeth Ashley, Rose McGready, Aung P Phyo, François Nosten, Tim J C Anderson

**Affiliations:** * Texas Biomedical Research Institute7620 NW Loop 410, San Antonio, TX, 78227, USA; † Faculty of Tropical Medicine, Mahidol UniversityBangkok, Thailand; ‡ Shoklo Malaria Research UnitMaesot, Thailand; § Centre for Tropical Medicine and Vaccinology, Churchill HospitalOxford, UK

**Keywords:** Population genetics – effective population size, empirical, genotypic richness, inbreeding, multiple-genotype infections, *Plasmodium falciparum*, recombination

## Abstract

Pathogen control programs provide a valuable, but rarely exploited, opportunity to directly examine the relationship between population decline and population genetics. We investigated the impact of an ∼12-fold decline in transmission on the population genetics of *Plasmodium falciparum* infections (*n* = 1731) sampled from four clinics on the Thai–Burma border over 10 years and genotyped using 96 genome-wide SNPs. The most striking associated genetic change was a reduction in the frequency of infections containing multiple parasite genotypes from 63% in 2001 to 14% in 2010 (*P* = 3 × 10^−15^). Two measures of the clonal composition of populations (genotypic richness and the β-parameter of the Pareto distribution) declined over time as more people were infected by parasites with identical multilocus genotypes, consistent with increased selfing and a reduction in the rate at which multilocus genotypes are broken apart by recombination. We predicted that the reduction in transmission, multiple clone carriage and outbreeding would be mirrored by an increased influence of genetic drift. However, geographical differentiation and expected heterozygosity remained stable across the sampling period. Furthermore, *N*_*e*_ estimates derived from allele frequencies fluctuation between years remained high (582 to ∞) and showed no downward trend. These results demonstrate how genetic data can compliment epidemiological assessments of infectious disease control programs. The temporal changes in a single declining population parallel to those seen in comparisons of parasite genetics in regions of differing endemicity, strongly supporting the notion that reduced opportunity for outbreeding is the key driver of these patterns.

## Introduction

The central aim of infectious disease control programs is to reduce the size of circulating pathogen populations. These control efforts provide a valuable opportunity to study the associated changes in pathogen population genetics. Changes in genetic diversity and population structure resulting from population decline are of central interest to conservation geneticists (Schwartz *et al*. 2007; Osborne *et al*. 2010). While the aims of pathogen control and conservation are diametrically opposite, pathogen systems can provide a valuable opportunity to understand the population genetic consequences of diminished population size. Pathogen systems may be particularly valuable in this respect, because generation times are short, genomes tend to be small and well characterized, and control programs in multiple countries allow replicated measurement of the genetic changes associated with control. Understanding the genetic changes associated with diminished population size in pathogen populations may also provide valuable metrics for monitoring the success of control efforts, if population genetic parameters accurately reflect transmission intensity.

*Plasmodium falciparum*, the causative agent of the most severe form of human malaria, is an obligately sexual hermaphrodite protozoan parasite. Haploid parasites replicate mitotically in the human host, with some parasite cells differentiating into male and female sexual stages (gametocytes). Male and female gametes fuse in the mosquito midgut to form a short-lived diploid zygote (ookinete), which then undergoes meiosis to generate haploid infective stages. Recombination occurs during the brief obligately sexual stage in the mosquito and results in the re-assortment of genes and generation of new parasite genotypes. *Plasmodium falciparum* has a mixed mating system. When male and female gametes of the same genotype fuse (self-fertilization), the haploid infective stages generated are unchanged by recombination, while when two genetically distinct gametes fuse (outbreeding), the genome of infective stages is reshuffled. The degree of outbreeding and recombination is thought to be determined by the proportion of people harbouring infections containing more than one parasite genotype (MIs). This varies considerably between populations, and scales with the level of malaria transmission (Anderson *et al*. 2000). In regions of intense malaria transmission, *P. falciparum* exhibits a predominantly outbred population structure characterized by extensive recombination, many MIs and few repeatedly sampled multilocus genotypes. In contrast, in low transmission regions, this parasite shows a largely clonal population structure characterized by high levels of self-fertilization, limited recombination, few MIs and identical genotypes found in multiple hosts (Conway *et al*. 1999; Anderson *et al*. 2000). Genetic diversity is also reduced in parasite populations showing low transmission, but it is unclear whether this is a reflection of demography or population history, because low transmission is observed in South American and SE Asian populations, and these populations are derived from an African source population.

In the past decade, there has been unprecedented increase in initiatives and resources aimed at malaria control (World Health Organization 2010) and renewed political will to fight malaria (Roll Back Malaria 2000). These have led to significant reductions in the numbers of malaria cases and malaria-related deaths in several countries (O'Meara *et al*. 2010; World Health Organization 2011). We focused on a region of low parasite transmission along the border between Thailand and Burma. Multiple lines of evidence suggest that transmission of *Plasmodium falciparum* malaria has declined significantly over the past decade in this region (V. I. Carrara, K. M. Lwin, A. P. Phyo *et al*., unpublished data). This is most likely due to sustained treatment of malaria patients with artemisinin combination therapy. This study was designed to examine changes in parasite population genetic parameters associated with reduced transmission in *P. falciparum*-infected patients sampled over 10 years in this region. We determined whether reductions in parasite transmission are associated with measurable changes in the carriage of multiple-genotype infections, genetic diversity, inbreeding, geographical structure and short-term effective population size (*N*_*e*_).

## Materials and methods

### Study sites and sampling

We collected filter paper blood spots from 1731 *Plasmodium falciparum* malaria patients attending four clinics spanning a 100-km region of the Thai–Burma border from 2001 to 2010. These samples were collected as part of a longitudinal trial evaluating parasite clearance rates in patients treated with various artemisinin-based combination therapies (Phyo *et al*. 2012).

We used two parameters to measure transmission intensity across the 10-year sampling period. First, we examined incidence of malaria infections in pregnant women attending weekly antenatal clinics. Blood smears were taken at each consultation regardless of whether the women were symptomatic. Second, we examined the proportion of *P. falciparum* malaria consultations among children <5 years old presenting to the four clinics. The data from pregnant women provide the most reliable indicator of transmission intensity because malaria status is determined during regular antenatal clinics, whereas children visit the clinics only when they are ill. Incidence data for other host age groups were not available.

### SNP genotyping

DNA was extracted from filter paper blood spots taken at admission from each patient using a two-step protocol to maximize DNA yield. Blood was first eluted from the filter paper using the GenSolve kit (GenVault Corporation), followed by DNA extraction using 96-well QIAamp 96 DNA Blood kits (Qiagen). We used the Illumina GoldenGate platform to genotype all infections at 96 single nucleotide polymorphisms (SNPs) distributed across all 14 chromosomes of the *P. falciparum* genome (Fig. S1, Table S1, Supporting information) (Phyo *et al*. 2012). The SNPs were selected using the PlasmoDB version 6.2 at http://www.plasmodb.org and were chosen because they are highly polymorphic in parasites from the Thai–Burma border and provide clearly scorable genotype data. We avoided SNPs in genes encoding surface proteins (vars, rifins, surfins and stevors) and transporters, as well as SNPs in telomeric genes, because these show extensive sequence variation and may be under strong selection, SNP genotyping was carried out according to the Illumina GoldenGate assay instructions except we used 100 ng DNA (containing an estimated 5–10 ng parasite DNA), rather than 250 ng starting DNA. Parasite DNA from the *P. falciparum* laboratory strain, 3D7, was used as a positive control in each 96-well plate genotyping run.

### Identification of multiple-genotype infections

Because blood stage malaria parasites are haploid, we expect to see only one allele per locus if an infection contains a single parasite clone and multiple alleles if more than one clone is present. Infections showing heterozygous base calls at >5% of the genotyped SNPs were considered MIs. We used a conservative 5% threshold to allow for the fact that small numbers of SNPs may be misscored as heterozygotes even in monoclonal infections.

### Relationships among parasite genotypes

We examined the relatedness among infections containing single genotypes by computing the number of alleles shared (*ps*) in pairwise comparisons and clustering parasites based on the distance metric *1-ps* using PHYLIP (Felsenstein 1993). Parasite genotypes from different patients that were identical at all SNPs examined were assumed to be identical by descent. To examine the power of our SNP markers to detect unique multilocus parasite genotypes (MLGs), we resampled different subsets of our SNPs and plotted the relationship between numbers of SNPs scored and numbers of MLGs identified. This resampling approach was implemented in GenClone v.2.0 (Arnaud-Haond & Belkhir 2007).

### Temporal and spatial distribution of identical MLGs

We conducted four analyses: (i) Probability of sampling identical MLGs. We measured the relationship between the time between two samples and the probability of finding identical MLGs within these samples. This analysis was conducted using the clonal subrange analysis implemented in GenClone v.2.0 (Arnaud-Haond & Belkhir 2007). We compared this relationship in infections from 2001–2004 and 2007–2010. We also estimated how long each MLG persists before being broken apart by recombination by tracking the earliest and latest sampling dates for infections bearing identical MLGs. (ii) Genotypic Richness. We used the genotypic richness index, *R*, to examine changes in the distribution of MLGs over time. *R* measures the proportion of unique genotypes present in the samples and is estimated as: *R = (G − 1)/(N − 1)* where *G* is the number of distinct genotypes and *N* is the sample size (Dorken & Eckert 2001). (iii) Pareto distributions. The frequency distribution of MLGs into different size classes conforms to the classical power law and is most appropriately approximated by the Pareto distribution (Arnaud-Haond *et al*. 2007). It is highly skewed with a large number of rare MLGs and a few common ones. We compared steepness of the Pareto slopes for frequency distributions in 2001–2004 and 2007–2010 to seek evidence of a change in distribution of MLGs. (iv) Sampling considerations. We evaluated the impact of sampling density on measures of both the genotypic richness (*R*) and the slope of the Pareto distribution (β) by resampling different numbers of infections (*n* = 100–1100) from the complete data set and recalculating these statistics.

### Multilocus linkage disequilibrium

We compared multilocus linkage disequilibrium (multilocus LD) in infections collected in 2001–2004 and 2007–2010. Multilocus LD was measured using the statistic I_A_S (standardized index of association), which compares the observed variance in numbers of alleles shared between parasites with that expected when alleles at different loci show no association (Haubold & Hudson 2000). To test the prediction that identical MLGs are the major source of LD in these parasite infections, LD estimations were performed with and without repeated MLGs.

### Genetic diversity and population structure

We assessed genetic diversity using Nei's expected heterozygosity (*H*_*E*_) index (Nei 1978). We compared genetic differentiation between Mawker–Thai and Maela Camp in the early (2001–2004) and late (2007–2010) sampling periods to seek evidence of increased population subdivision over time. Measures of genetic differentiation (*F*_ST_ values) (Weir & Cockerham 1984) were derived using FSTAT software (Goudet 1995).

### Effective population size

We used the SNP data from single-genotype infections to examine changes in short-term *N*_*e*_ estimates over the 10-year sampling period. We used fluctuation in allele frequencies between adjacent years to estimate short-term (Variance) effective population size (*N*_*e*_*V*) using the pseudo-maximum likelihood method implemented in the program MLNE v.1547 (Wang 2001) and the temporal-based moments method (Waples 1989) implemented in the NeEstimator software (Peel *et al*. 2004). *N*_*e*_*V* estimates assumed a 2-month generation time for *P. falciparum*.

## Results

### Declining incidence of *P. falciparum* malaria on the Thai–Burma border

**Fig. 1:**
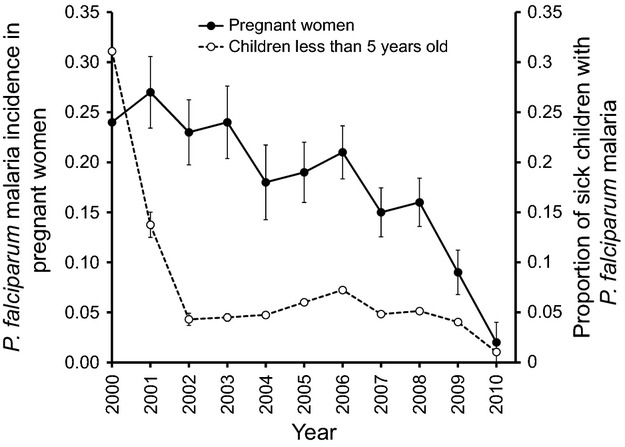
Decline in the incidence of *Plasmodium falciparum* malaria on the Thai–Burma border. The incidence of *P. falciparum* malaria in pregnant women attending antenatal clinics and the proportion of falciparum malaria consultations among sick children <5 years old were measured. Error bars represent 95% confidence intervals. Both measures decreased significantly over time.

Our collaborators at the Shoklo Malaria Research Institute measured trends in the incidence of malaria on the Thai–Burma border from 2000 to 2010 using data from 90 188 children <5 years old and 13 508 pregnant women (Table S2, Supporting information). Their data reveal that the incidence of *Plasmodium falciparum* malaria decreased from 0.24 infections per person per year in 2000 to 0.02 per person per year in 2010 in pregnant women. Similarly, the proportion of *P. falciparum* malaria consultations among sick children <5 years old decreased progressively from 33% in 2000 to 1% in 2010 (Fig. 1). Epidemiology data indicating declining transmission (V. I. Carrara, K. M. Lwin, A. P. Phyo *et al*., unpublished data) will be presented in detail elsewhere.

### Summary statistics for genetic data

We prepared and genotyped DNA from 1731 infections (Table S3, Supporting information). These included 629 from Wang Pha, 396 from Maela, 146 from Mae Kon Ken and 560 from Mawker–Thai. Maela and Mawker–Thai were sampled across the 10-year period, while samples were only available for Wang Pha and Mae Kon Ken from 2007 to 2010 (Table 1). Data from three loci were difficult to score and were excluded. All samples were successfully scored at an average of 99.6% of the 93 genotyped SNPs (range, 92.5–100%). We genotyped the laboratory parasites 3D7, Dd2, W2 or HB3 on each genotyping run. No discrepant genotype calls were observed in 26 control genotypes (26 × 93 = 2418 SNP calls). One SNP call in the 2418 SNP calls in the controls was erroneously scored as heterozygous. This gives an error rate of 4.13 × 10^−4^ for misscoring SNPs as heterozygous in single-genotype infections. We conducted analyses either by year or by comparing early (2001–2004) and late (2007–2010) sampling periods. Sampling was very sparse in 2005–2006, so these years were not included in the later analyses. All 93 loci were polymorphic with minor allele frequency ranging from 0.1 to 0.5. Mean *H*_*E*_ was 0.427 ± 0.075 (SD) in the early period compared with 0.429 ± 0.075 (SD) in the late time period (Mann–Whitney *U*-test; *U* = 4175, *Z* = −0.409, *P* = 0.6828).

**Table 1:** Summary data for *Plasmodium falciparum* infections examined from the four clinics

	All locations			Mawker–Thai			Maela camp			Mae kon Ken			Wang Pha		
					
Year	*N*	*H*_*E*_	freq [MIs]	*N*	*H*_*E*_	freq [MIs]	*N*	*H*_*E*_	freq [MIs]	*N*	*H*_*E*_	freq [MIs]	*N*	*H*_*E*_	freq [MIs]
2001	78	0.43 ± 0.01	0.63 ± 0.05	15	—	—	63	0.43 ± 0.01	0.73 ± 0.06	—	—	—	—	—	—
2002	197	0.43 ± 0.01	0.51 ± 0.04	115	0.43 ± 0.01	0.50 ± 0.05	81	0.43 ± 0.01	0.52 ± 0.06	—	—	—	1	—	—
2003	184	0.42 ± 0.01	0.45 ± 0.04	107	0.43 ± 0.01	0.37 ± 0.05	76	0.41 ± 0.01	0.54 ± 0.06	1	—	—	—	—	—
2004	101	0.43 ± 0.01	0.39 ± 0.05	31	0.42 ± 0.01	0.29 ± 0.08	51	0.42 ± 0.01	0.45 ± 0.07	—	—	—	19	—	—
2005	30	0.42 ± 0.01	0.30 ± 0.08	26	0.42 ± 0.01	0.27 ± 0.09	4	—	—	—	—	—	—	—	—
2006	14	—	—	14	—	—	—	—	—	—	—	—	—	—	—
2007	45	0.42 ± 0.01	0.29 ± 0.07	5	—	—	6	—	—	4	—	—	30	0.42 ± 0.01	0.34 ± 0.09
2008	549	0.42 ± 0.01	0.25 ± 0.02	112	0.42 ± 0.01	0.21 ± 0.04	53	0.43 ± 0.01	0.30 ± 0.06	68	0.42 ± 0.01	0.16 ± 0.05	316	0.41 ± 0.01	0.28 ± 0.03
2009	356	0.42 ± 0.01	0.28 ± 0.02	92	0.42 ± 0.01	0.24 ± 0.04	35	0.43 ± 0.01	0.26 ± 0.07	55	0.41 ± 0.01	0.28 ± 0.06	174	0.42 ± 0.01	0.31 ± 0.04
2010	177	0.42 ± 0.01	0.14 ± 0.03	43	0.41 ± 0.01	0.14 ± 0.05	27	0.41 ± 0.01	0.15 ± 0.07	18	—	—	89	0.42 ± 0.01	0.14 ± 0.04

*H*_*E*_ was calculated from single-genotype infections only. *H*_*E*_ and the frequency of MIs (freq [MIs]) were calculated only when *N* > 20.

### Temporal changes in multiple-genotype infections

**Fig. 2:**
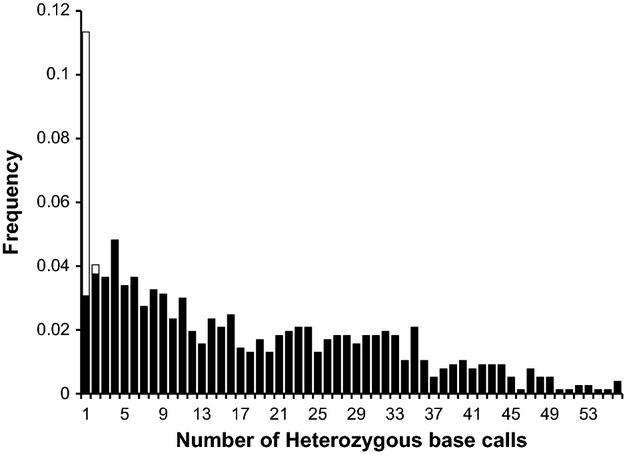
Frequency distribution of heterozygous SNP calls. Most commonly observed were samples with just 1 heterozygous SNP, suggesting that many of these may result from genotyping error rather than true MIs. The white bars show the expected proportions of heterozygous SNP calls due to genotyping error, calculated from the observed miscalling rate of 4.13 × 10^−4^ in our single-genotype controls. With this calling error rate, we seldom expect >3 SNPs per sample to be misscored as heterozygous.

**Fig. 3:**
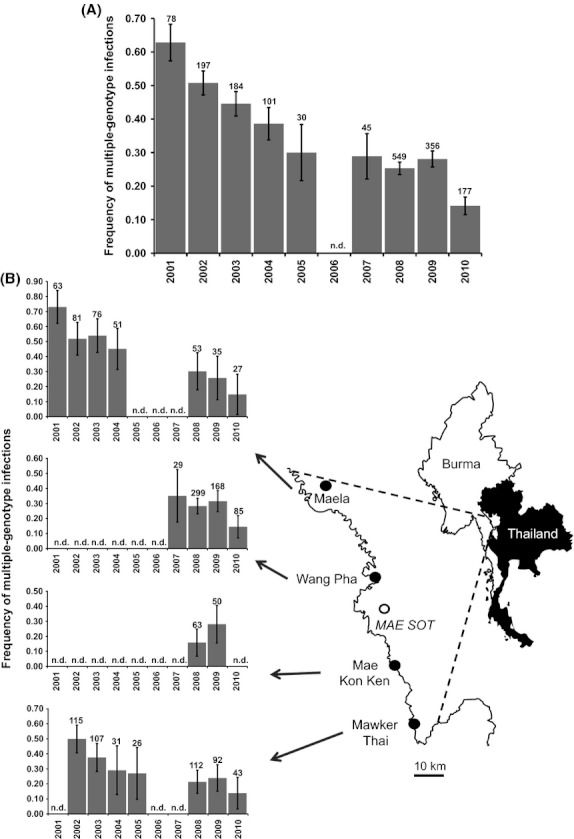
Decline in the frequency of multiple-genotype infections. We examined temporal changes in the frequency of multiple-genotype infections (MIs) at all clinics combined (A) and at individual clinics (B). Error bars are 95% confidence intervals. The frequency of MIs decreased significantly over time, consistent with reduced transmission. Data were not plotted when *n* < 20 for a particular year. The map of Thailand and Burma shows the border region of Tak Province (white shading) where clinics are located. The expanded map shows the position of the four clinics along the border. Mae Sot, the main town in this region, is shown for reference.

Of the 1731 infections genotyped, 767 had multiple alleles at one or more of the 93 loci (Fig. 2). Given the error rate (4.13 × 10^-4^), small numbers (1–3) of heterozygous SNPs are likely to be scoring errors. A total of 558 infections showed multiple alleles at >5 loci and were classified as MIs. The remaining 1173 infections contained a single predominant genotype and were classified as single-genotype infections. There was a marked decline in the frequency of MIs from 63% in 2001 to 14% in 2010 (Chi-square test; χ^2^ = 62.329, d.f. = 1, *P* = 3 × 10^−15^) (Fig. 3A). Trends in the frequency of MIs at individual clinics mirrored that of all clinics combined (Fig. 3B). The decline in MI carriage was robust to changes in the threshold used for defining MIs and was observed using thresholds ranging from >1 to >25 loci showing multiple alleles (Fig. S2, Supporting information). Multiple logistic regression analysis showed that sampling year was strongly associated with carriage of MIs (Table 2). Location and admission parasitaemia were also associated with carriage of MIs, but there were no significant associations with patient age and sex (Table 2). In addition, carriage of MIs was not associated with severe malaria infections that required blood transfusion or rescue therapy (Table 2).

**Table 2:** Logistic regression analysis of factors associated with the carriage of multiple-genotype infections (MIs)

Variable	Odds ratio	Likelihood ratio χ^2^	*P*-value
Sampling year	1.207 [1.153–1.264]	67.822	1.79 × 10^-16^ ***
Parasite density	1.257 [1.086–1.454]	10.656	0.001 ***
Age	0.997 [0.989–1.006]	3.286	0.070
Sex: Male	1.040 [0.835–1.295]	0.122	0.727
Rescue treatment	0.964 [0.732–1.269]	0.070	0.791
Blood transfusion	0.834 [0.574–1.211]	0.904	0.341
Location	Compared with Mawker–Thai	15.832	0.001 ***
Maela Camp	0.612 [0.462–0.811]	—	—
Wang Pha	0.672 [0.496–0.909]	—	—
Mae Kon Ken	0.963 [0.596–1.555]	—	—
Season	Compared with Jan–Mar	1.114	0.774
April–June	0.910 [0.671–1.234]	—	—
July–September	1.061 [0.747–1.505]	—	—
October–December	0.950 [0.686–1.317]	—	—

Figures in square brackets represent 95% CIs. We used the likelihood ratio test to determine whether the exclusion of a particular variable significantly reduces the goodness of fit of the regression model. The likelihood ratio χ^2^ and its associated *P*-value are derived from comparing the full model and the constrained model without the variable in question. Significant values are indicated by asterisks (***). Carriage of MIs is associated with lower admission parasite densities and decreases significantly over time.

### Temporal change in composition of MIs

**Fig. 4:**
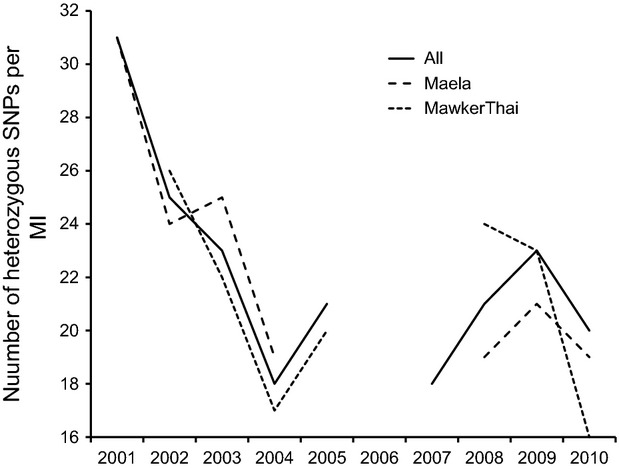
Change in composition of multiple infections over time. We plotted the mean numbers of heterozygous SNPs within MIs over time in all sites combined and in the two sites (Mawker–Thai and Maela) sampled across the whole time period. Years in which < 9 MIs were available were excluded. The distribution of numbers of heterozygous SNPs per MI is shown in Fig 2.

The number of heterozygous SNPs within multiple-genotype infections can provide an indirect measure of the relatedness between parasites or the number of clones within MIs. We examined the mean number of heterozygous SNP calls in 558 MIs (>5 SNPs with multiple alleles) over time (Fig. 4). Numbers of heterozygous SNPs/MI dropped significantly from 31 ± 15 (SD) in 2001 to 20 ± 11 (SD) in 2010 at all locations combined. Linear regression analysis showed that sampling year was the only factor significantly associated with the decline in numbers of heterozygous SNPs within MIs (*F* = 6.097, d.f. = 1, *P* = 0.014). The decline in numbers of heterozygous SNPs within MIs was also significantly associated with sampling year at Maela (*F* = 5.220, d.f. = 1, *P* = 0.006) but not at Mawker–Thai (*F* = 0.311, d.f. = 1, *P* = 0.733).

### Temporal and spatial distribution of identical multilocus genotypes

**Fig. 5:**
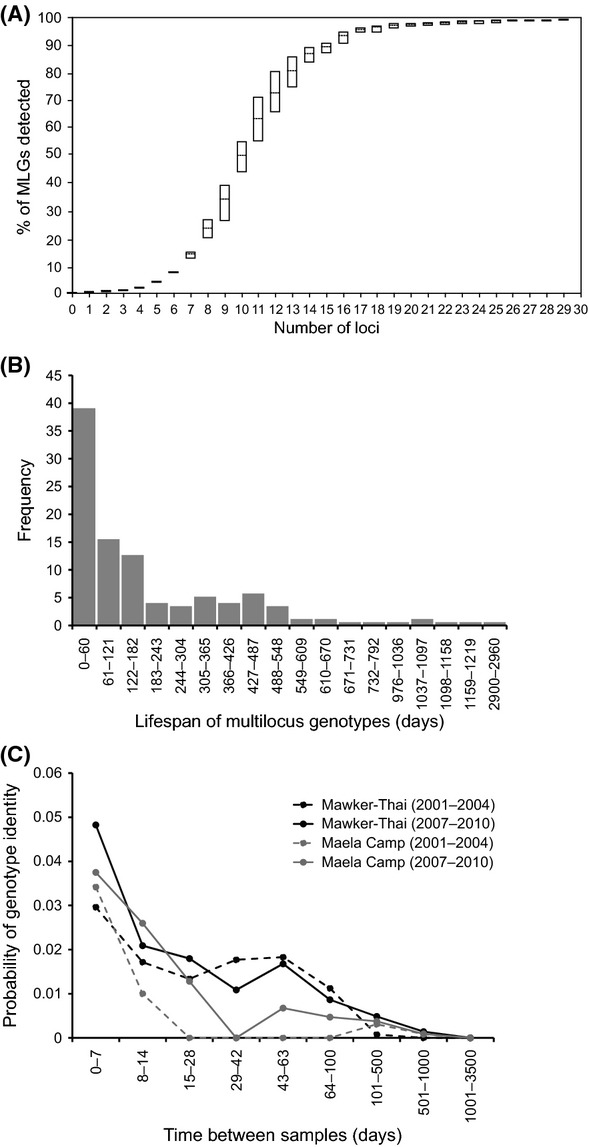
Detection and distribution of MLGs over time. (A) Resolution power of SNPs for unique multilocus genotypes (MLGs). A resampling approach was used to determine the minimum number of SNP markers required to exhaustively identify the distinct parasite MLGs within infections. The edges of the boxes show the minimum and maximum percentage of MLGs detected, and the central line shows the average percentage of MLGs identified using the number of markers shown on the x-axis. After 25 loci, the percentage of unique MLGs identified plateaus. Therefore, the 93 SNPs used have more than sufficient discriminatory power for all the MLGs in infections. (B) Persistence of identical multilocus genotypes: Over 60% of MLGs infecting multiple patients passed through several generations in the mosquito without being broken apart by genetic recombination. The median duration for MLGs was 91 days (range, 1–2938 days). (C) Probability of sampling identical MLGs. We examined the probability of sampling identical MLGs within infections sampled at different time intervals. There was a small increase in the probability of genotype identity between 2001–2004 and 2007–2010. The same MLG was sampled <6% of the time in infections collected <28 days apart.

We conducted a resampling analysis to determine the power of our SNP set to identify MLGs. This analysis determined that a minimum of 25 SNP markers were required to exhaustively identify all distinct MLGs present in infections (Fig. 5A) and is consistent with previous findings (Daniels *et al*. 2008). Therefore, the 93 SNPs used provided more than sufficient resolution power for the identification of MLGs in these infections.

We predicted that decreased transmission and diminishing numbers of infections containing multiple genotypes would increase parasite inbreeding, as well as propagation and long-term stability of identical MLGs. We identified 174 MLGs infecting between 2 and 15 people over the ten-year period (Fig. S3, Supporting information). Most (76%) of the genotypes were only seen at a single location. While some genotypes were ephemeral, others persisted for up to 8 years without being broken apart by recombination (Fig. 5B). The median lifespan of these MLGs was 91 days (range, 1–2938 days). Identical MLGs tended to be clustered in time. The probability of sampling identical MLGs was highest (0.03–0.05) for samples collected within the same week and declined as time between samples increased. There was an increased probability of sampling identical MLGs within infections collected <14 days apart in 2007–2010 compared with infections sampled in 2001–2004 at both Maela Camp and Mawker–Thai (Fig. 5C).

### Changes in distribution of MLGs

**Fig. 6:**
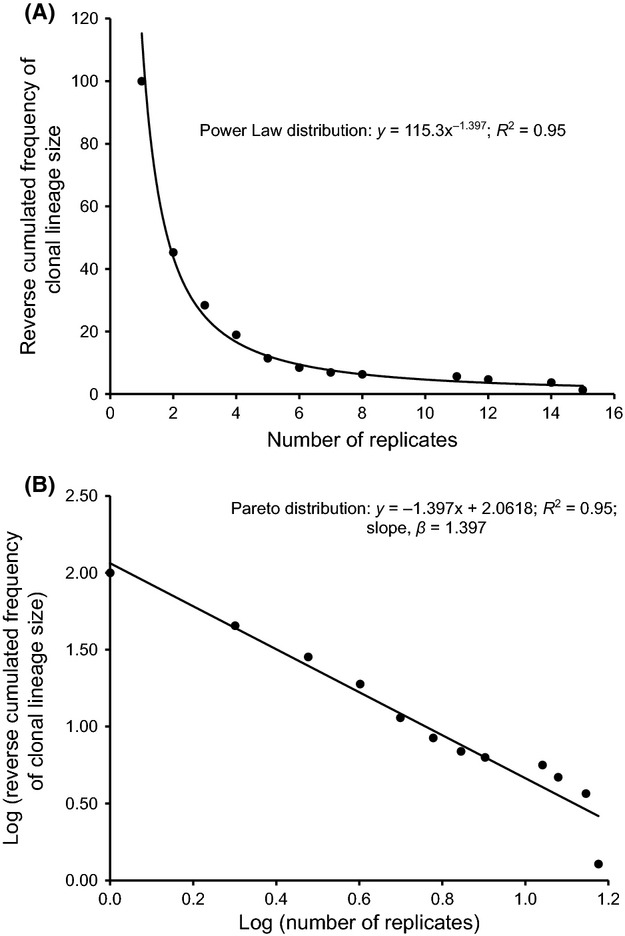
Calculation of Pareto slopes (A) Frequency distribution of MLG abundance. Using the complete data set, we plotted the frequency of MLG abundance. This fits with a power law, with many rare MLGs and smaller number of common MLGs. (B) Measurement of β. Log transformation of both axes generates a straight line relationship from which goodness of fit and the slope (β) are calculated.

**Fig. 7:**
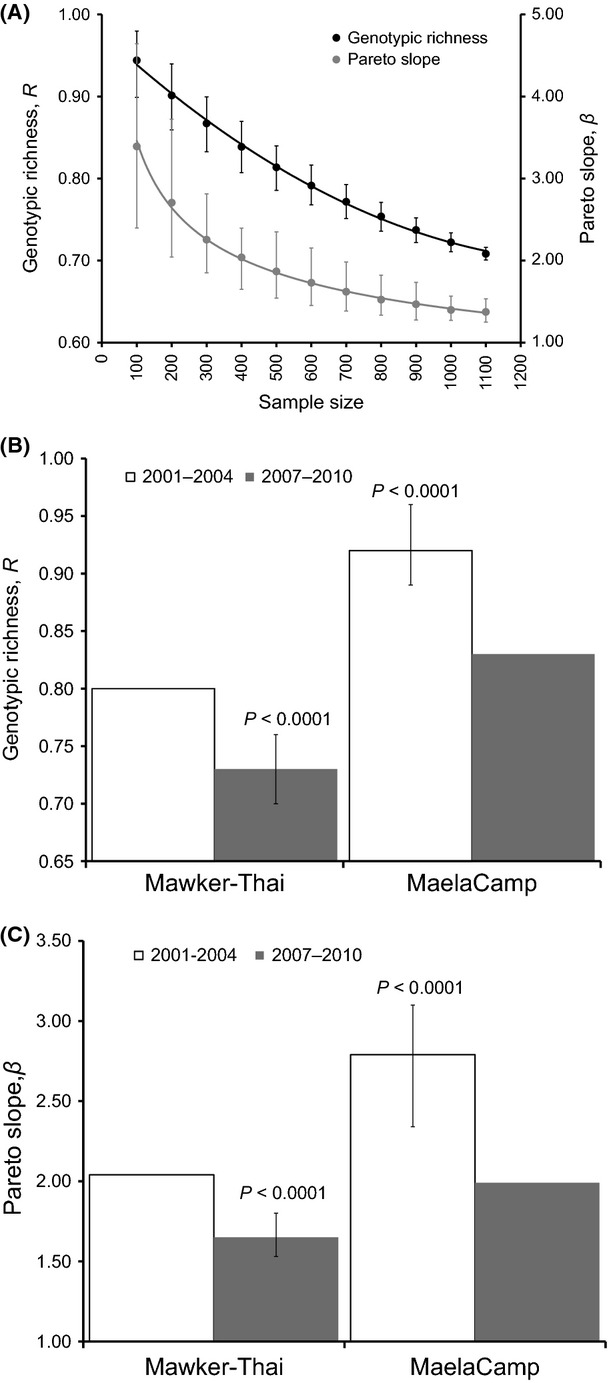
Analysis of genotype diversity indices. (A) Effect of sampling density on genotypic diversity indices. We investigated the effect of sampling effort on estimates of the genotypic richness index, R, and the slope of the Pareto distribution, β, using randomly sampled infections. Both R and β were strongly influenced by sample size, indicating that incorrect inferences about genetic diversity can be reached if sampling density is not standardized. Estimates of R (B) and β (C) obtained with resampled data of equal sample size show a significant decline in both indices between 2001–2004 and 2007–2010. Significance was assessed by comparing values of R and β in the less sampled period with 10 000 resampled subpopulations of equal size from the more densely sampled period.

We predicted that reduction in malaria transmission would be accompanied by change in the distribution and abundance of MLGs, measured by the genotypic richness index, R, and the slope of the Pareto distribution, β. The frequency distribution of MLGs into different size classes followed the typical power law (Fig. 6A), approximated by the Pareto distribution (Fig. 6B). Because genotypic diversity indices may be affected by sampling density, we evaluated the effect of sampling effort on empirical measures of R and β. We found both indices to be strongly influenced by sampling density (Fig. 7A). To avoid potential sampling bias in our R and β comparisons, we used a resampling approach to equalize sample sizes to derive and compare empirical estimates of R and β for infections collected in 2001–2004 and 2007–2010. Estimates of both R and β decreased significantly over time at both Mawker–Thai and Maela Camp (Fig. 7B,C) after correction for sample density.

### Multilocus linkage disequilibrium

We found higher values of I_A_S suggesting increasing LD from 2001–2004 to 2007–2010 at two locations where we had dense sampling during the two time periods. I_A_S estimates increased from 0.0040 to 0.0109 and 0.0025 to 0.0056 at Mawker–Thai and Maela Camp, respectively. We were concerned that sampling effort might influence our I_A_S estimates. Therefore, we resampled the data 100 times to equalize sample sizes for the two time periods. The mean I_A_S estimate increased significantly (*P* < 0.01) from 0.0040 in 2001–2004 to 0.0109 ± 0.0019 (95% CI) in 2007–2010 at Mawker–Thai but remained unchanged at Maela Camp (I_A_S = 0.0024 in 2001–2004 versus 0.0025 ± 0.0010 (95%CI) in 2007–2010 (*P* = 0.43). I_A_S measures obtained without repeated MLGs also showed a significant increase in LD at Mawker–Thai (I_A_S = 0.0014 in 2001–2004 versus 0.0047 ± 0.0006 (95% CI) in 2007–2010 (*P* < 0.01).

### Population differentiation

We measured *F*_ST_ between the two clinics with dense sampling in both the early and late sampling periods. *F*_ST_ was 0.007 ± 0.0.019 (SD) in 2001–2004 compared with 0.013 ± 0.025 (SD) in 2007–2010. The change was not significant (Wilcoxon signed rank test; *Z* = −1.021, *P* = 0.3073).

### Effective population size

We predicted that reduction in the incidence of *P. falciparum* malaria (a measure of population census size) might be mirrored by reduction in short-term effective population size (*N*_*e*_*V*) of the parasite population. *N*_*e*_*V* estimates ranged from 582 to ∞ using MLNE or from 43 to 328 using the moments' estimator (Table 3). The two measures showed a nonsignificant correlation (*r*^2^ = 0.394, *P* = 0.1311). There was no evidence for a substantial reduction in *N*_*e*_*V* over time or of a positive relationship between measures of malaria incidence in pregnant women and *N*_*e*_*V* estimates derived using the temporal-based moments estimator (*r*^2^ = 0.197, *P* = 0.2706). Furthermore, contrary to expectation, there is a negative relationship between malaria incidence and the maximum likelihood estimator of *N*_*e*_*V* (*r*^2^ = 0.670, *P* = 0.0243). Estimates of *N*_*e*_*V* using population samples spaced at 2- or 3-year intervals are consistent with analyses of consecutive years showing either increasing (MNLE) or stable (moments) *N*_*e*_*V* estimates (Table 3).

**Table 3:** Temporal changes in short-term effective population size of *Plasmodium falciparum* on the Thai–Burma border

Temporal comparison	Maximum likelihood_*N*_*e*_	Moments *N*_*e*_
Consecutive years		
2001–2002	∞ [208–∞]	161 [82–383]
2002–2003	703 [323–3582]	143 [87–236]
2003–2004	582 [301–1647]	99 [61–161]
2004–2005	837 [295–∞]	43 [26–72]
2007–2008	2694 [957–∞]	98 [58–167]
2008–2009	2709 [1681–4636]	328 [206–514]
2009–2010	2645 [1539–5195]	212 [132–335]
Two-year intervals		
2001–2003	986 [335–∞]	216 [129–362]
2004–2006	1071 [329–∞]	73 [43–127]
2007–2009	2049 [916–∞]	196 [115–340]
Three-year intervals		
2001–2004	572 [272–2536]	218 [133–357]
2004–2007	818 [419–2499]	161 [99–261]
2007–2010	2776 [1109–∞]	349 [199–658]

Figures in square brackets represent 95% CIs. Temporal comparisons 2005–2006 and 2006–2007 are not shown because of the small sample size (14) in 2006. Estimates are derived from the combined population sample from all four clinics. Individual analyses of Mawker–Thai and Maela, the clinics sampled across the 10-year period, showed similar patterns.

## Discussion

Reduced census population size is expected to result in increased inbreeding, diminished heterozygosity, increased geographical differentiation and reduced short-term *N*_*e*_*V*. We examined the genetic changes that accompany successful reduction in malaria transmission as measured by the number of cases during routine surveillance of pregnant women in antenatal clinics. We observed a reduction in the proportion of infections containing multiple parasite genotypes. This was accompanied by an associated reduction in two measures of the distribution of clonally identical parasite genotypes (genotype richness and the β parameter of the Pareto distribution), consistent with increased levels of inbreeding. However, we did not observe changes in the level of genetic drift, as reflected by changes in expected heterozygosity, geographical structure and short-term *N*_*e*_.

### MIs and transmission intensity

**Fig. 8:**
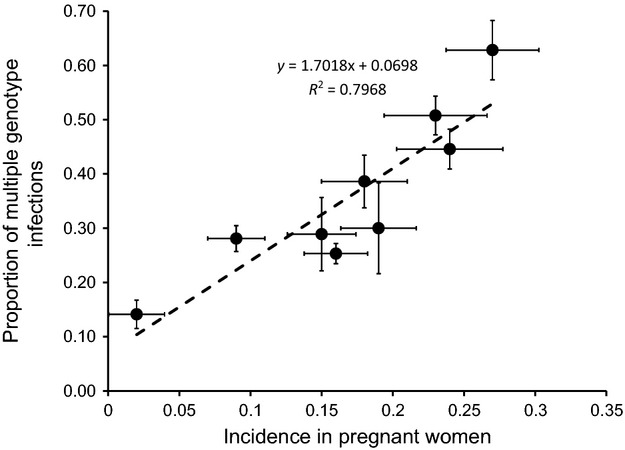
Relationship between malaria transmission and MI carriage. Malaria incidence (cases per person per year) plotted against MI carriage for years 2001–2010 (2006 data were excluded because only 14 samples were genotyped). The error bars are 1 SD for both *x* and *y* variables.

Reduced transmission was accompanied by a significant decrease in the frequency of MIs from 63% in 2001 to 14% in 2010. These longitudinal trends are consistent with findings from cross-sectional studies examining malaria parasite genetics in regions of differing endemicity in both humans (Konate *et al*. 1999; Anderson *et al*. 2000; Bendixen *et al*. 2001; Mobegi *et al*. 2012) and lizards (Vardo & Schall 2007). While MIs predominate in regions of intense malaria transmission, they tend to be less frequent in low transmission regions. Our data show a strong linear relationship between incidence of malaria in pregnant women and MI carriage (*r*^2^ = 0.8), indicating a 17% increase in MI carriage for an increase in incidence of 0.1 cases per person per year (Fig. 8). The observed 50% reduction in MI carriage rates, and the linear association between transmission intensity and MI carriage in this study, suggests that carriage of MIs can be a useful genetic indicator of transmission intensity. This measure will be particularly useful in low transmission areas (<1 infective bite per person per year) such as SE Asia, because the proportion of people infected with MIs saturates when transmission is at quite moderate levels (>10 infective bites per year) (Anderson *et al*. 2000).

One simple interpretation of the decline in MI carriage assumes that MIs result from bites from 2 or more infected mosquitoes (superinfection). Under this assumption, the trends observed reflect a reduction in the number of people exposed to bites from more than one infective mosquito. However, recent data question the validity of the superinfection model. Analyses of the component clones within multiple-genotype infections demonstrate that MIs consist predominantly of related parasites that are likely to result from haploid recombinant infective stages inoculated by single mosquito bites rather than superinfection (Nkhoma *et al*. 2012). The data in this study also provide support for single mosquito inoculation as the main source of MIs. *Plasmodium falciparum* is transmitted by forest dwelling mosquitoes (*A. minimus*, *A. dirus* and *A. maculatus*), on the Thai–Burma border and much of SE Asia. As a consequence, adult men (>15 years) working in the forests are exposed to infective mosquito bites more than women or children. This is reflected by the fact that >15-year-old males make up 55% of all malaria patients, outnumbering >15-year-old females by 3.5-fold and <5-year-old children of both sex by 19-fold (Fig S4, Supporting information). Despite the disparity in malaria exposure across age and sex classes, there was no detectable influence of age or sex on MI carriage in a multiple regression analysis (Table 2). Similarly, season did not influence MI carriage, despite the fact that risk of malaria varies considerably by ∼4-fold between low and high transmission seasons (October–December versus April–June). Hence, the proportion of people infected with MIs is most likely to reflect the number of mosquitoes infected with >1 parasite genotypes rather than the number of people exposed to superinfection.

The composition of multiple-genotype infections also changes over time. Numbers of heterozygous SNPs scored in MIs declined over the sampling period (Fig. 4). This trend may be explained by two processes. First, the parasite genotypes within multiple infections may be becoming more closely related. For example, increased similarity among parasites within infection is predicted if MIs are serially transmitted between hosts (Nkhoma *et al*. 2012) as might be expected when transmission diminishes. Second, these trends are consistent with a decrease in the mean numbers of clones within MIs over time.

### Increase in inbreeding with declining transmission

Reduction in population size is expected to increase inbreeding levels because the probability of mating between related individuals is increased. This effect may be magnified in *Plasmodium*, because populations of sexual stages are subdivided among mosquitoes, and the size of subpopulations is strongly associated with transmission. Hence, Wahlund effects (Wahlund 1928) might be expected to increase inbreeding when transmission declines. Direct measurement of outbreeding is possible in *P. falciparum* by genotyping oocysts dissected from the mosquito midgut (Annan *et al*. 2007). However, in South-East Asia, <1 in 10 000 mosquitoes may carry malaria parasites (Imwong *et al*. 2011) so in practice, this is not feasible. We therefore inferred changes in inbreeding rates by examining the number and distribution of identical 93-locus genotypes in blood stage parasites. Both indices examined—genotypic richness index (R) and the slope of the Pareto distribution (β)—demonstrated a change in the proportion of patients infected with the same parasite genotype, and a change in the distribution of clonal group sizes within populations. These two parameters have been recommended for quantifying clonal population structure because they are minimally affected by sampling density (Arnaud-Haond *et al*. 2007). However, in this longitudinal data set, we observed a strong sampling effect (Fig 7A). Genotypic richness declined from 0.94 to 0.71 when we randomly subsampled 100–1100 parasites from the complete data set. Similarly, β declined from 3.39 to 1.37 as sample density increased. Dependence on sampling density occurs because identical 93-locus genotypes are strongly clustered in time and space. Identical 93-locus genotypes were most frequently sampled <14 days apart, and probabilities rapidly declined after this (Fig. 5C). Hence, sparse sampling tends to recover less identical genotypes than dense sampling. Similarly, spatial clustering is evident in these data as 76% of identical 93-locus genotypes were specific to one of the four clinics. Using a resampling approach to equalize sample sizes in 2001–2004 and 2007–2010, we found that both R and β decreased significantly over time (Fig. 7B,C) at both Mawker–Thai and Maela. Once again, the observed decrease in R and β, as transmission declines on the Thailand–Burma border, corresponds to differences observed between locations with differing endemicity. For example, identical parasites are rarely observed in different patients in sub-Saharan Africa where transmission is high, but are extremely common in South American countries where transmission is very low (Anderson *et al*. 2000). However, dependence on sampling density and the problem of detecting identical genotypes when MIs predominate make these metrics less useful than the proportion of MIs for assessing transmission intensity.

The large number of longitudinal sample genotypes from a single geographical region allows us to examine the length of time that multilocus genotypes persist without being broken apart by recombination. The median persistence time (91 days) is equivalent to two generations if we assume a ∼8 generations/year. One MLG persisted for 2938 days (∼65 generations) without being broken apart by genetic recombination. The long lifespan of MLGs suggests that there are fewer opportunities for recombination in this parasite population and is consistent with declining levels of MIs. We observed a small increase in the probability of sampling identical MLGs within infections sampled 28 days apart between 2001–2004 and 2007–2010. Sampling of identical MLGs may impair our ability to distinguish reinfections from treatment failures, leading to the overestimation of treatment failures in antimalarial drug efficacy trials (Snounou & Beck 1998). This can occur if a patient is reinfected with parasites that are genetically identical to those from a previous infection. However, because this probability remained below 6%, it is unlikely to have significant influence on estimates of drug efficacy in this setting.

### Why no detectable change in *N*_*e*_?

Despite substantial reduction in transmission, and strong indirect evidence for increased inbreeding rates, there was no significant decline in genetic diversity, in geographical structure or in effective population size (*N*_*e*_*V*), estimated from fluctuation in allele frequencies between years (Table 3). This result is surprising, but similar observations have been made in other systems. For example, Teacher *et al*. (2009) observed an 83% reduction in frog population size following infection with ranavirus, while Queney *et al*. (2000) observed a 90% reduction in rabbit populations following a viral epizootic. In neither study, were significant changes in *N*_*e*_*V* observed. There are two possible explanations for our failure to detect a decline in *N*_*e*_. First, there may be reductions in *N*_*e*_, but our analysis lacks sufficient power to detect these patterns. Temporal methods for measurement of *N*_*e*_ are most useful when *N*_*e*_ is quite small (<100) and allele frequency fluctuations can be easily detected. When true *N*_*e*_ is large (>100), fluctuations in allele frequency may be very small, so enormous samples sizes are required to measure *N*_*e*_*V* with accuracy (Hare *et al*. 2011). Hence, this result may be a false negative resulting from insufficient sampling. Second, there may be no change in *N*_*e*_ despite the fact that transmission is significantly reduced. This counterintuitive result could be explained in two ways:

1. The Thai–Burma border forms the edge of a large focus of endemic *P. falciparum* malaria stretching across Burma and into Southern China and Bangladesh. Burma alone contains >40 million people at risk of *P. falciparum*, with >200 000 confirmed cases per year (World Health Organization 2011). Hence, while transmission has been reduced locally in the study area, *N*_*e*_ may remain large because this is only one small corner of an extensive region where transmission remains high. There is extensive movement of migrant workers and refugees across the Thai–Burma border (V. I. Carrara, K. M. Lwin, A. P. Phyo *et al*., unpublished data), so there is likely to be extensive exchange of parasites and parasite genes linking populations of *P. falciparum* in Thailand and Burma.
2. Measures of short-term *N*_*e*_*V* estimate the harmonic mean population size and are therefore closest to minimum rather than maximum estimates. Malaria transmission is strongly seasonal with the main transmission season in April–June. While the data demonstrate ˜4-fold increase in parasite population census size during the peak transmission season, numbers of malaria parasites surviving between seasons may be more critical for determining *N*_*e*_. We therefore examined relationship between *N*_*e*_ and transmission in October–December, when transmission is lowest in the year. However, once again there was no positive association between the levels of malaria transmission in children and maximum likelihood *N*_*e*_ (*r*^2^ = 0.333, *P* = 0.1747) as well as moments *N*_*e*_ (*r*^2^ = 0.573, *P* = 0.0296).

### Utility of genetic measures for assessing transmission intensity

Direct measurement of malaria transmission intensity is expensive, extremely labour intensive and subject to error. Therefore, in malaria control programmes, most resources go towards rapid detection and treatment of cases rather than to unbiased cross-sectional surveys of disease incidence or measurement of entomological inoculation rates (Kelly-Hope & McKenzie 2009). Population genetic measures that allow indirect assessment of transmission intensity and the efficacy of interventions could therefore be particularly useful (Volkman *et al*. 2012). Our analyses show that both the proportion of MIs and the proportion of unique malaria genotypes (measured by genotype richness or β parameter of the Pareto distribution) declined with decreasing transmission in this study. There are advantages and disadvantages to both types of measurement. The proportion of MIs has advantages because it is unbiased by spatial or temporal sampling, uses data from all patients and is remarkably constant among different age and sex classes of the host population despite considerable variation in transmission rate among host subgroups. On the negative side, the proportion of MIs is dependent on the sensitivity of genotyping methods used and is therefore difficult to compare between studies. Genotype richness and the β parameter of the Pareto distribution can only be measured using single-genotype infections, where haplotypes can be accurately described and are influenced by patterns of spatial and temporal sampling. However, assuming sufficient markers is used to identify unique haplotypes, these statistics will be minimally affected by genotyping methodology. We anticipate that combining multiple measures of transmission including epidemiological and serological markers (Cook *et al*. 2011) and population genetic measures will provide the most effective approach to monitoring transmission decline following intervention.

## Notes

S.C.N., F.N. and T.J.C.A. designed the research; S.C.N., S.N., S.A-S., E.A., R.M. and A.P.P. performed the research; S.C.N., F.N. and T.J.C.A. analysed the data, S.C.N. and T.J.C.A. wrote the paper.

## Data accessibility

Assay details for GoldenGate genotyping of SNPs: uploaded as online supporting information (Table S1, Supporting information). Malaria transmission data set: uploaded as online supporting information (Table S2, Supporting information). SNP data set for 1731 infections: uploaded as online supporting information (Table S3, Supporting information).

## Supporting information

**Tables S1** (GoldenGate SNP assay details) and Table S3 (SNP dataset analyzed) are provided separately as tab delimited text files.

**Table S2** Epidemiology of *P. falciparum* in children and pregnant women.

**Fig. S1** GoldenGate SNP genotyping. Shown here is a typical SNP graph for the A/G SNP on chromosome 14 position 1853537, showing clustering of infections based on their genotypes. Genotype calls are based on the normalized ratio of fluorescence intensity signals for the “A” and the “G” allele in the sample.

**Fig. S2** Influence of thresholds used to call MIs. To further determine if the trends observed are robust to the threshold used for categorizing MIs, infections were categorized as MIs using four different threshold values (>1, >5, >10 and >25 heterozygous SNP calls).

**Fig. S3** UPGMA tree showing clustering of multilocus genotypes (MLGs) based on the proportion of shared alleles.

**Fig. S4** Disparity in malaria in malaria cases among age and sex classes.
