# Supplementary material for: Population genetic correlates of declining transmission in a human pathogen
Source: Mol Ecol. 2012 Nov 2;22(2):273–85. doi: 10.1111/mec.12099 (PMC3537863; doi:10.1111/mec.12099)
Supplement: Supplementary file 1 [file mec0022-0273-SD1.pdf]

Supplementary online material

Population genetic correlates of declining transmission in a human pathogen

Standwell C. Nkhoma[1], Shalini Nair[1], Salma Al-Saai[1], Elizabeth Ashley[2,3,4], Rose McGready[3], Aung Pyae Phyo[3], François Nosten[2,3,4] and Tim J.C. Anderson[1*]

[1]Texas Biomedical Research Institute, San Antonio, Texas, USA, [2]Faculty of Tropical Medicine, Mahidol University, Bangkok, Thailand, [3]Shoklo Malaria Research Unit, Maesot, Thailand, [4]Centre for Tropical Medicine and Vaccinology, Churchill Hospital, Oxford, UK

Contents:

Figures S1-S4

Table S2 (Malaria epidemiology)

Tables S1 (GoldenGate SNP assay details) and Table S3 (SNP dataset analyzed) are provided separately as tab delimited text files.

Figure S1

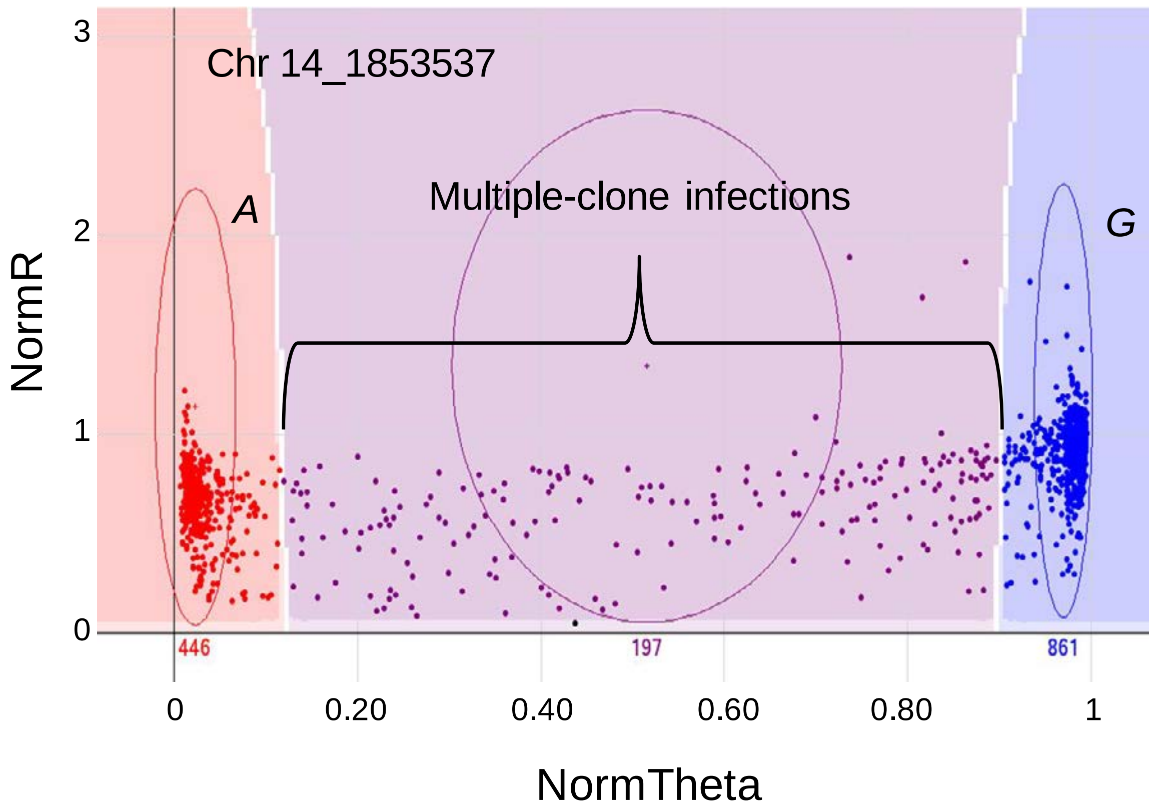

Figure S1. GoldenGate SNP genotyping. Shown here is a typical SNP graph for the A/G SNP on chromosome 14 position 1853537, showing clustering of infections based on their genotypes. Genotype calls are based on the normalized ratio of fluorescence intensity signals for the “A” and the “G” allele in the sample. Multiple-genotype infections appear as mixtures (heterozygotes) in the middle cluster.

Figure S2

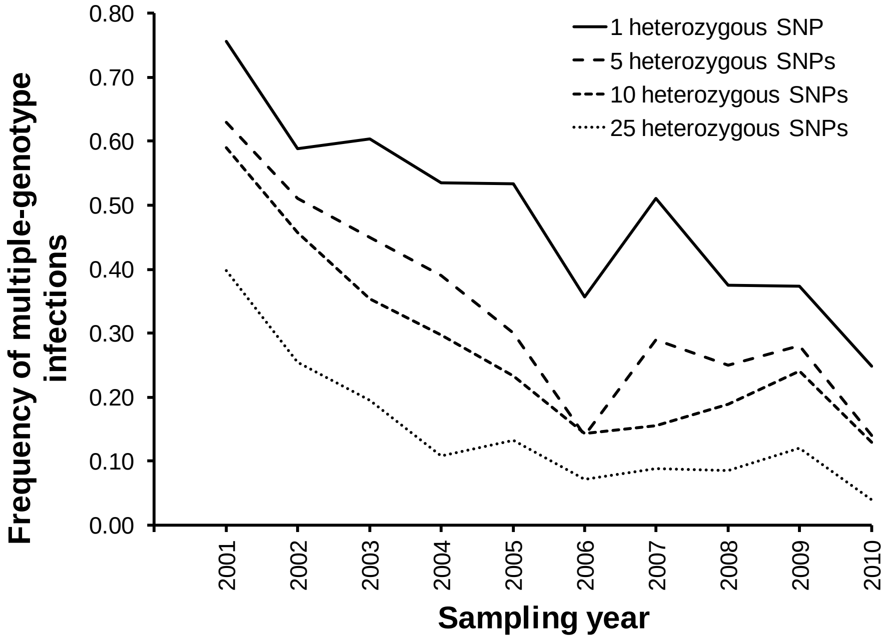

Fig S2. Influence of thresholds used to call MIs. To further determine if the trends observed are robust to the threshold used for categorizing MIs, infections were categorized as MIs using 4 different threshold values (>1, >5, >10 and >25 heterozygous SNP calls). The decline in MI carriage is observed at all threshold values, demonstrating that the trends observed are robust.

Figure S3

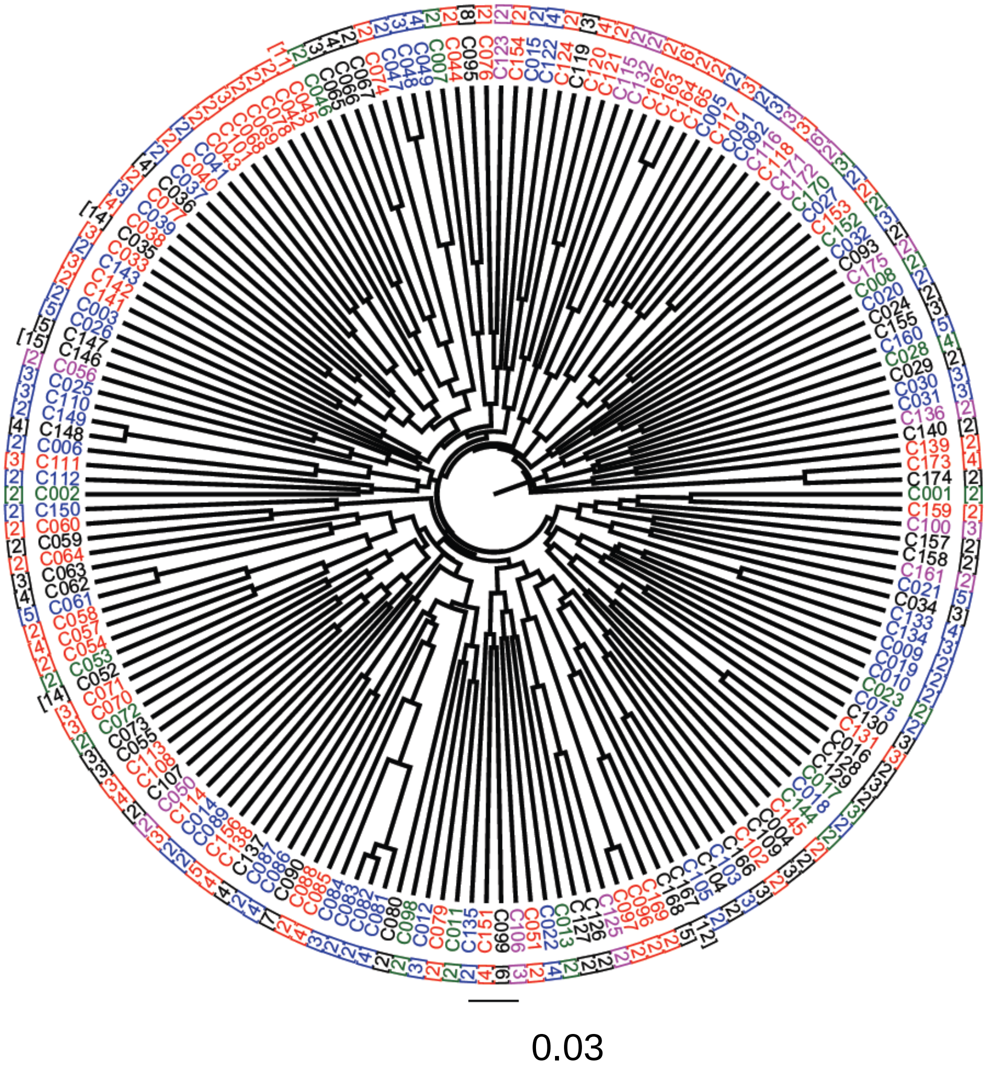

Figure S3. UPGMA tree showing clustering of multilocus genotypes (MLGs) based on the proportion of shared alleles. Tip labels represent the identification codes for MLGs, the different color schemes are used to identify individual clinics where the MLGs were recovered (blue = Mawker-Thai; green = Maela Camp; purple = Mae Kong Khen; red = Wang Pha; black = recovered from > 1 location). Square brackets contain the number of patients harboring identical MLGs. There is strong spatial clustering of identical MLGs with 76% of MLGs localized to only one clinic.

Figure S4

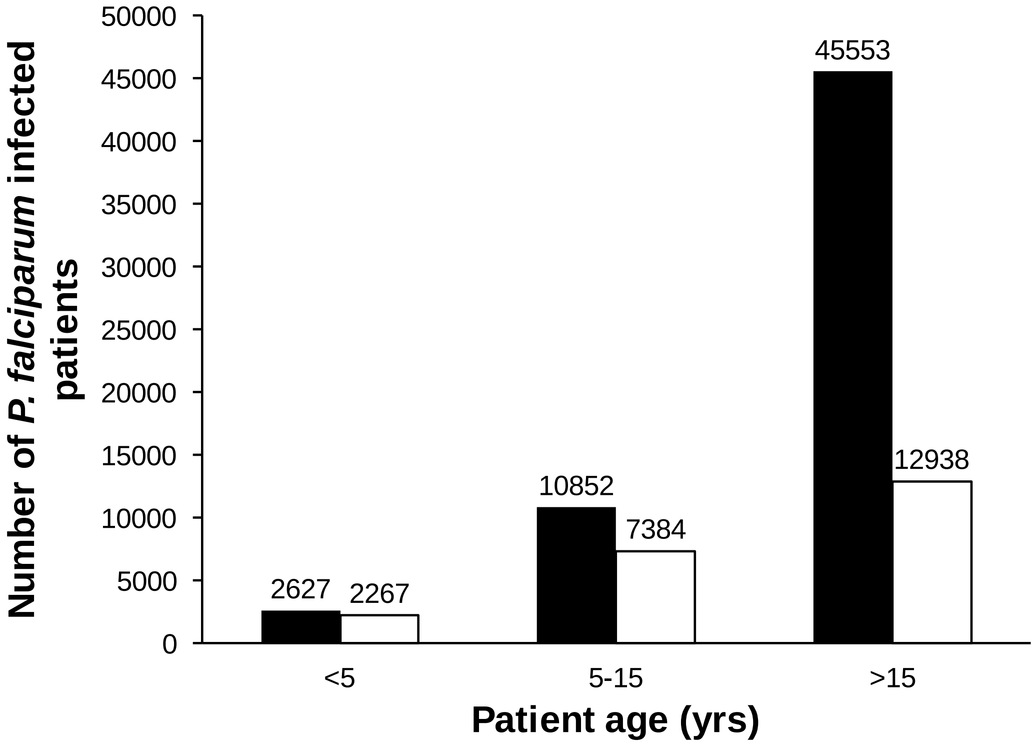

Figure S4. Disparity in malaria in malaria cases among age and sex classes. These data show the breakdown of 81621 *P. falciparum* malaria infections visiting the Southern clinics between 2001-2010 and the northern clinics between 2004-2010 by age group and host gender. The disparity in cases across age and gender remains extremely stable over the 10 year period. Data from Carrara et al (submitted).

Table S2. Epidemiology of *P. falciparum* in children and pregnant women. Presence of parasites were determined by rapid diagnosis tests (Paracheck, Malariascan, or Optimal) and malaria blood smear. Full details are provided in Carrara et al (submitted)

**Year**	**No. of children (<5 yrs) examined**	**No. of *P. falciparum* cases**	**No. of pregnant women examined**	**No. of *P. falciparum* cases**
2000	1344	423	588	142
2001	3205	497	679	186
2002	3182	336	593	135
2003	3756	269	550	131
2004	5902	313	900	163
2005	9617	583	1151	219
2006	12032	883	1297	277
2007	13641	667	1495	219
2008	12700	669	1648	270
2009	12062	500	2326	203
2010	12747	177	2281	51
TOTAL	90188	5317	13508	1996
