# Supplementary figures and images for: Population genetic correlates of declining transmission in a human pathogen
Source: Mol Ecol. 2012 Nov 2;22(2):273–85. doi: 10.1111/mec.12099 (PMC3537863; doi:10.1111/mec.12099)

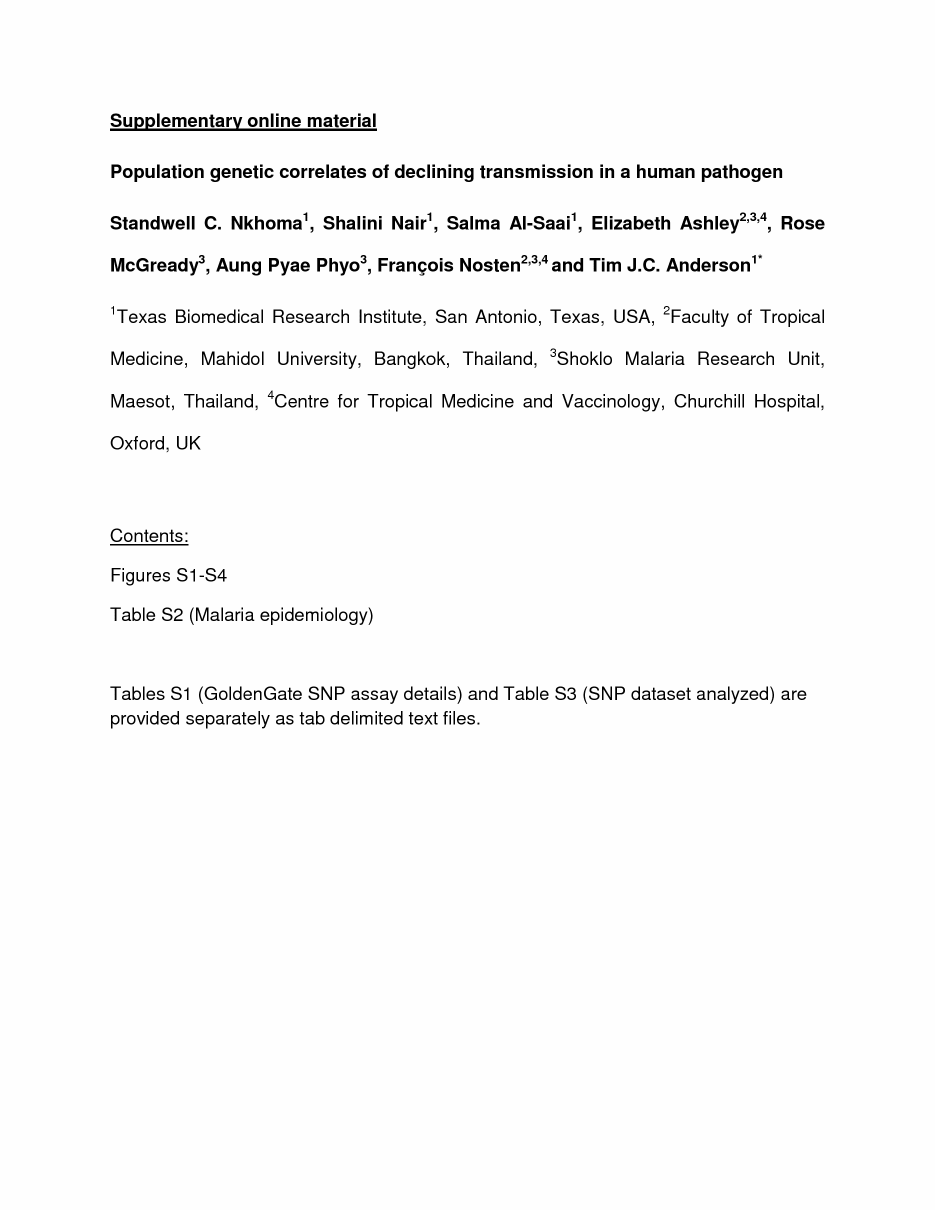

Supplement: Supplementary file 2 [file mec0022-0273-SD2.png]
